# Food insecurity and cardiovascular disease risk factors among U.S. adults

**DOI:** 10.1186/s12889-025-22031-9

**Published:** 2025-02-28

**Authors:** Michael F. Royer, Lisa G. Rosas, Abby C. King

**Affiliations:** 1 Stanford Prevention Research Center, Stanford University School of Medicine, Palo Alto, CA USA; 2 Department of Epidemiology and Population Health, Stanford University School of Medicine, Stanford, CA USA

**Keywords:** Food insecurity, Cardiovascular disease, Blood pressure, Cholesterol, Diabetes, Smoking, Sedentary, Body mass index, Gender, Race, Ethnicity, NHANES

## Abstract

**Background:**

Food insecurity is an ongoing public health issue that involves limited or uncertain access to food. National rates of food insecurity among U.S. households have increased from 10.2% (~ 13,500,000 households) in 2021 to 12.8% (~ 17,000,000 households) in 2022 and most recently 13.5% (~ 18,000,000 households) in 2023. Food insecurity is associated with various health conditions and behaviors that raise the risk of cardiovascular disease (CVD). Little is known about whether the associations between food insecurity and CVD risk factors among U.S. adults differ by gender and race/ethnicity.

**Methods:**

The 2017–2018 National Health and Nutrition Examination Survey dataset was accessed for a cross-sectional study on the associations between food insecurity and six CVD risk factors among U.S. adults. Food insecurity was measured with the 10-item Adult Food Security Survey Module. General linear models were used to analyze the associations between food insecurity and high blood pressure, high cholesterol, diabetes, cigarette smoking, sedentary time, and body mass index (BMI). Moderation analyses examined differences in the association between food insecurity and CVD risk factors by gender and race/ethnicity.

**Results:**

Food insecurity was positively associated with high blood pressure (B = 0.104, *p* = 0.04), diabetes (B = 0.087, *p* = 0.006), and cigarette smoking (B = 0.239, *p* = 0.002); and food insecurity was negatively associated with sedentary time (B=-0.765, *p* = 0.02). Moderation analyses for gender revealed that women who were food insecure had greater BMI than men who were food insecure (B = 2.284, *p* = 0.02). Moderation analyses for race/ethnicity determined that, compared to White adults with food insecurity, high blood pressure rates were lower among Asian (B=-0.164, *p* = 0.04) and Black (B=-0.153, *p* = 0.04) adults with food insecurity, high cholesterol rates were greater among Latino adults with food insecurity (B = 0.168, *p* = 0.04), and cigarette smoking rates were lower among Black (B=-0.246, *p* = 0.03) and Latino (B=-0.380, *p* = 0.006) adults with food insecurity.

**Conclusions:**

This research produced useful insight into how food insecurity relates to conditions and behaviors that increase CVD risk. Evidence from this study can inform both longitudinal and prevention efforts aimed at investigating food insecurity as both a harmful social determinant of health and a potential predictor of CVD risk among U.S. adults.

**Trial registration:**

N/A.

## Background

Food insecurity is an ongoing public health issue that comprises limited or uncertain access to sufficient food [1]. Adults in the U.S. are increasingly experiencing this harmful social determinant of health as food insecurity rates have steadily increased nationally from 10.2% (~ 13,500,000 households) in 2021 to 12.8% (~ 17,000,000 households) in 2022 and most recently rose to 13.5% (~ 18,000,000 households) in 2023 [2, 3]. Gender disparities in food insecurity were detected among U.S. adults without children in 2023 with approximately 16.2% of women and 14.0% of men having been food insecure [3]. Wide racial/ethnic disparities in food insecurity were also reported in 2023 as non-Hispanic Black (23.3%) and Hispanic (21.9%) individuals had much higher rates of food insecurity than non-Hispanic White (9.9%) individuals [3]. Public perception in 2024 has pointed to persistent food-price inflation and the end of COVID-19 economic supports as primary reasons for heightened U.S. food insecurity [4]. 

Several types of barriers to food access act as primary contributors to food insecurity. Barriers to accessing food typically include insufficient finances [5], lack of transportation [6], poor food preparation and cooking skills [7], and inadequate information about food assistance resources [8]. In addition to the health risks posed by limited access to food, various unhealthy diet-related behaviors that occur during food insecurity experiences include disordered eating [9], low fruit and vegetable intake [10], and high consumption of ultra-processed foods (i.e., containing high quantities of various artificial ingredients) and sugar-sweetened beverages [11, 12]. Food insecurity is associated with myriad diet-related chronic diseases such as cardiovascular disease (CVD) [13], a condition that is present among approximately 48% of adults (~ 121,500,000 individuals) in the U.S [14]. 

CVD can pertain to one of several conditions including arrhythmia, coronary artery disease (CAD), heart disease, heart attack, heart failure, and stroke [15]. CAD, which consists of the buildup of plaque in the heart’s arteries (i.e., atherosclerosis) [16], is the most common type of CVD in the U.S. and was responsible for an estimated 371,506 deaths in 2022 [17]. Heart disease, a catch-all term for an array of heart ailments (e.g., atherosclerotic CVD, cardiomyopathy, coronary heart disease, etc.) [18], has been the leading cause of death among U.S. adults for over 100 years [19] and most recently accounted for 702,880 deaths in 2022 [17]. An assortment of poor health outcomes and behaviors act as risk factors for the development or exacerbation of CVD [17]. CVD risk factors that consist of ongoing health hazards include conditions like high blood pressure [20], high LDL cholesterol [21], obesity [22], and diabetes [23]. Behavioral risk factors of CVD include habits and behaviors that are unhealthy when done in excess such as physical inactivity [24], poor nutrition [25], excessive alcohol use [26], and tobacco smoking [27]. 

Emerging evidence has shown food insecurity to be associated with many of the same health outcomes (e.g., diabetes, obesity) and behaviors (e.g., smoking, alcohol misuse) that increase the risk of CVD [28]. An observational study on food insecurity and obesity among a large sample of U.S. adults determined that women who were food insecure had a higher prevalence of obesity than their food secure counterparts while no differences in obesity were detected between men who were food insecure or food secure [29]. Results from a meta-analysis of 18 research articles totaling over 55 million pooled participants reported that household food insecurity was associated with an increased odds of type-2 diabetes [30]. Regarding behavioral CVD risk factors, findings from observational research among U.S. adults indicated that food insecurity was associated with a decreased odds of meeting physical activity guidelines but was not associated with sedentary time [31]. Separately, outcomes from an observational study among midwestern U.S. adults suggested that food insecurity was associated with an increased odds of cigarette smoking, heavy alcohol use, and dual substance use [32]. 

Much is still uncertain concerning the association between food insecurity and CVD risk factors. Contradictory findings from various studies have been reported for the association between food insecurity and high cholesterol. One study found that food insecurity was linked to high cholesterol in women but not men [33], another study determined that adults with food insecurity had higher odds of high cholesterol [34], and a third study found no relationship between food insecurity and total cholesterol levels [35]. While a few investigations on health disparities among U.S. adults have reported how the association between food insecurity and CVD prevalence may differ by race/ethnicity [36], no known studies have examined whether race/ethnicity moderates the associations between food insecurity and the CVD risk factors of high cholesterol and sedentary time. Additional knowledge gaps include the extent to which gender moderates the associations between food insecurity and these two less investigated CVD risk factors in the field of food insecurity research. In light of ongoing health disparities and health equity gaps in the U.S., there is an urgent need to ascertain whether the association between food insecurity and CVD risk factors differ by gender and race/ethnicity.

Therefore, the objectives of this cross-sectional study were to determine whether (1) food insecurity was positively associated with six unique CVD risk factors, (2) gender moderated the associations between food insecurity and the CVD risk factors, and (3) race/ethnicity moderated the associations between food insecurity and the CVD risk factors. The American Heart Association reported that the incidence of CVD begins steadily increasing in U.S. adults starting at age 40 [37], so it is critical that knowledge gaps regarding the association between food insecurity and CVD risk factors are addressed for this age range. Examining the multifaceted relationship between food insecurity and CVD can inform evidence-based prevention efforts aimed at both food insecurity as a harmful social condition and CVD as the leading cause of mortality among U.S. adults.

## Methods

### Study design

This cross-sectional study involved accessing the publicly available National Health and Nutrition Examination Survey (NHANES) 2017–2018 archival dataset from the Centers for Disease Control and Prevention’s (CDC) to analyze the association between food insecurity and six major CVD risk factors among U.S. adults aged 40 years and older.

### Participant sample

The NHANES respondents included in this study were a nationally representative sample of U.S. adults ages 40 years and older (*n* = 3676). Using NHANES survey weights, it was estimated that the complex survey design translated into an approximated total study sample of 144,819,356 individuals. Consent to participate was not required since the deidentified 2017–2018 NHANES dataset was accessed from the CDC’s publicly available data archive.

### Outcome measures

The independent variable of food insecurity was measured using the United States Department of Agriculture’s (USDA) validated 10-item Adult Food Security Survey Module (AFSSM) [38]. The 10 items from the Adult FSSM include the following: In the last 12 months… (1) I worried whether my food would run out before I got money to buy more. (2) The food that I bought just didn’t last and I didn’t have money to get more. (3) I couldn’t afford to eat balanced meals. (4) Did you ever cut the size of your meals or skips meals because there wasn’t enough money for food? (5) How often did this happen? (6) Did you ever eat less than you felt you should because there wasn’t enough money for food? (7) Were you ever hungry but didn’t eat because there wasn’t enough money for food? (8) Did you lose weight because there wasn’t enough money for food? (9) Did you ever not eat for a whole day because there wasn’t enough money for food? (10) How often did this happen?

Affirmative responses (e.g., Often True, Sometimes True, Yes, Almost Every Month, Some Months but Not Every Month, Only 1 or 2 Months) for each of the 10 AFSSM items were summed and totaled to determine participant food security scores. Respondents were classified as having full food security with a score of 0, marginal food security with a score of 1–2, low food security with a score of 3–5, and very low food security with a score of 6–10. Food security corresponded with scores indicating full food security or marginal food security. Food insecurity corresponded with scores indicating low food security or very low food security.

The dependent variables included six separate CVD risk factors that were all measured using self-report items within the NHANES survey [39]. High blood pressure was measured by asking respondents if they had ever been told by a doctor or other health professional that they had hypertension or high blood pressure (Yes, No). High cholesterol was measured by asking respondents if they had ever been told by a doctor or other health professional that their blood cholesterol level was high (Yes, No). Diabetes was measured by asking respondents if they had ever been told by a doctor or health professional that they have diabetes (Yes, No). Cigarette smoking was measured by asking respondents if they now smoke cigarettes (Every Day, Some Days, Not at All). Cigarette smoking responses of “Every Day” and “Some Days” were converted to “Yes”, and a response of “Not at All” was converted to “No”. Sedentary activity was measured by asking respondents how much time they usually spent sitting in a typical day (0–1320 min). Sedentary activity minutes were converted to hours by dividing the original sedentary activity data by 60 min (0–22 h). Body mass index (BMI) was measured by asking respondents for their body height (inches) and weight (pounds). Inches were converted to meters (MTR) by dividing the original height in inches by 39.37 (1 MTR = 39.37 inches). Pounds were converted to kilograms (KG) by dividing the original weight in pounds by 2.205 (1 pound = 2.205 KG). BMI was then calculated for each respondent by dividing their weight in KG by their height in MTR squared (BMI = KG / MTR^2^).

Participant characteristics were also collected by NHANES for both descriptive and covariate purposes. Age was measured by asking respondents for their current age in years (0–80). Gender was measured by asking respondents whether they were female or male. Race/ethnicity was measured by asking respondents whether they were Mexican American, Other Hispanic, Non-Hispanic White, Non-Hispanic Black, Non-Hispanic Asian, or Other Race including Multi-Racial. Mexican American and Other Hispanic were combined to create a Hispanic/Latino category. Income was measured by asking respondents whether their annual household income was $20,000 or More or Below $20,000. Education was measured by asking respondents if their highest grade or level of school completed or the highest degree received was Less than 9th Grade, 9th -11th Grade including 12th Grade with No Diploma, High School Graduate/GED or Equivalent, Some College or Associate’s Degree, or College Graduate or Above. A “Less than High School” category was created by combining “Less than 9th Grade” with “9th -11th Grade including 12th Grade with No Diploma”. Marital Status was measured by asking respondents whether they were Married, Widowed, Divorced, Separated, Never Married, or Living with a Partner. The Marital Status variable was made dichotomous by retaining the “Married” category and creating a “Not Married” category by combining Widowed, Divorced, Separated, Never Married, or Living with a Partner.

### Statistical analysis

RStudio packages including “stats”, “survey”, “svy”, and “glm” were utilized for study data analyses [40]. To account for the complex NHANES survey design and to ensure nationally representative estimates, all analyses incorporated NHANES sampling weights, strata, and primary sampling units, as recommended by the CDC [41]. Evaluation of the three research hypotheses involved the development of general linear models (GLM) to analyze the associations between the independent variable of food insecurity and the dependent variables of six CVD risk factors that included high blood pressure, high cholesterol, diabetes, cigarette smoking, sedentary time, and BMI.

Logistic regression analyses were used for GLM models with dichotomous dependent variables (high blood pressure, high cholesterol, diabetes, cigarette smoking). Linear regression analyses were used for GLM models with continuous dependent variables (sedentary time, BMI). Initial GLM models used bivariate regression analyses to determine the unadjusted association between food insecurity and each of the individual six CVD risk factors. Expanded GLM models then used multivariable regression analyses that similarly analyzed the association between food insecurity and each individual CVD risk factor with statistical adjustment for the covariates of age, gender, race/ethnicity, and income. Regression analyses were conducted using listwise deletion to handle missing data, ensuring that only cases with compete data for all variables in the model were included. Covariates used in the adjusted regression analyses were determined by multicollinearity tests across participant characteristics to avoid redundancy in the adjustment of potential confounders and to enhance the precision of statistical estimates.

To address the first hypothesis, GLM models using bivariate regression generated unadjusted estimates of the association between food insecurity and the six CVD risk factors. GLM models using multivariable regression then generated adjusted estimates of the association between food insecurity and the six CVD risk factors by statistically adjusting for the covariates of age, race/ethnicity, and income. To address the second hypothesis, an interaction term for gender and food insecurity was created and included as the independent variable within the adjusted GLM models. These moderation analyses included adjusted GLM models that used multivariable regression to generate estimates detailing how the association between food insecurity and the six CVD risk factors differed between male and female genders. To address the third hypothesis, an interaction term for race/ethnicity and food insecurity was created and included within the adjusted GLM models as the independent variable. These moderation analyses included adjusted GLM models that used multivariable regression to generate estimates detailing how the associations between food insecurity and the six CVD risk factors differed across Asian, Black, Latino, Other, and White racial/ethnic categories.

## Results

The U.S. adults ages 40 years and older (*n* = 3676) who were sampled for this study were an average age of 60.8 (SD = 11.9) years old. Adults in the sample (Table 1) were mostly women (51.1%), White (35.7%), married (55.4%), had completed some college (30.7%), and reported an annual income of $20,000 or more (72.2%).

**Table 1:** Characteristics of U.S. Adults ages 40 years and older (*n* = 3676) by food security status

Characteristics	Total (%)^b^	Food Secure (%)^c^	Food Insecure (%)^c^	No FS/FI (%)^a, c^
**Sample (n)**	*n* = 3676 (100)	*n* = 2724 (74.1)	*n* = 764 (20.8)	*n* = 188 (5.1)
**Age (Years)**	M = 60.8 SD = 11.9^a^	M = 61.5 SD = 12.1	M = 58.4 SD = 10.8	M = 61.0 SD = 11.8
**Gender**				
Women	1877 (51.1)	1381 (73.6)	397 (21.2)	99 (5.2)
Men	1799 (48.9)	1343 (74.7)	367 (20.4)	89 (4.9)
**Race/Ethnicity**				
Asian	511 (13.9)	436 (85.3)	42 (8.2)	33 (6.5)
Black	882 (24.0)	633 (71.8)	193 (21.9)	56 (6.3)
Hispanic/Latino	793 (21.6)	498 (62.8)	249 (31.4)	46 (5.8)
White	1314 (35.7)	1046 (79.6)	226 (17.2)	42 (3.2)
Other	176 (4.8)	111 (63.1)	54 (30.7)	11 (6.2)
**Income**				
Below $20,000	667 (18.1)	351 (52.6)	316 (47.4)	0 (0)
$20,000 or More	2653 (72.2)	2255 (85.0)	398 (15.0)	0 (0)
Missing Income	356 (9.7)	118 (33.1)	50 (14.1)	188 (52.8)
**Education**				
Less than HS^a^	799 (21.7)	483 (60.5)	274 (34.3)	42 (5.2)
HS^a^ Graduate	869 (23.6)	627 (72.2)	208 (23.9)	34 (3.9)
Some College	1128 (30.7)	848 (75.2)	224 (19.9)	56 (4.9)
College Graduate	868 (23.6)	762 (87.8)	55 (6.3)	51 (5.9)
Missing Education	12 (0.3)	4 (33.3)	3 (25.0)	5 (41.7)
**Marital Status**				
Married	2036 (55.4)	1612 (79.2)	315 (15.5)	109 (5.3)
Not Married	1635 (44.5)	1110 (67.9)	448 (27.4)	77 (4.7)
Missing MS	5 (0.1)	2 (40.0)	1 (20.0)	2 (40.0)

^a^HS = High School; M = Mean; MS = Marital Status; No FS/FI = Missing Food Security Data; SD = Standard Deviation

^b^Percentage across Participant Characteristic

^c^Percentage across Food Security Statuses by Participant Characteristic Group

The participant sample (Table 2) was mostly food secure (74.1%), had an average BMI of 29.2 (SD = 6.7), engaged in 5.6 (SD = 3.4) hours of sedentary time each day, did not smoke cigarettes (28.2%), did not have high blood pressure (50.5%), did not have high cholesterol (53.5%), and did not have diabetes (78.1%).

**Table 2:** Cardiovascular disease risk factors by food security status among U.S. Adults ages 40 years and older (*n* = 3676)

Risk Factors	Total (%)^b^	Food Secure (%)^c^	Food Insecure (%)^c^	No FS/FI (%)^a, c^
**Sample (n)**	*n* = 3676 (100)	*n* = 2724 (74.1)	*n* = 764 (20.8)	*n* = 188 (5.1)
**High BP** ^a^				
No	1855 (50.5)	1390 (74.9)	368 (19.8)	97 (5.3)
Yes	1813 (49.3)	1329 (73.3)	393 (21.7)	91 (5.0)
Missing BP^a^	8 (0.2)	5 (62.5)	3 (37.5)	0 (0)
**High Cholesterol**				
No	1965 (53.5)	1464 (74.5)	394 (20.1)	107 (5.4)
Yes	1675 (45.6)	1237 (73.9)	360 (21.5)	78 (4.6)
Missing CH^a^	36 (0.9)	23 (63.9)	10 (27.8)	3 (8.3)
**Diabetes**				
No	2873 (78.1)	2179 (75.8)	546 (19.0)	148 (5.2)
Yes	801 (21.8)	545 (68.0)	217 (27.1)	39 (4.9)
Missing Diabetes	2 (0.1)	0 (0)	1 (50)	1 (50)
**Smokes Cigs** ^a^				
No	1039 (28.2)	799 (76.9)	202 (19.4)	38 (3.7)
Yes	616 (16.8)	370 (60.1)	205 (33.3)	41 (6.6)
Missing Cigs^a^	2021 (55.0)	1555 (76.9)	357 (17.7)	109 (5.4)
**Sedentary Hours**	M = 5.6 95% CI = 5.4, 5.7	M = 5.7 95% CI = 5.6, 5.8	M = 4.9 95% CI = 4.7, 5.2	M = 5.7 95% CI = 5.2, 6.2
**BMI** ^a^	M = 29.2 95% CI = 28.9, 29.4	M = 28.9 95% CI = 28.7, 29.1	M = 30.3 95% CI = 29.8, 30.8	M = 29.2 95% CI = 28.2, 30.2

^a^BMI = Body Mass Index; BP = Blood Pressure; CH = Cholesterol; CI = Confidence Interval; Cigs = Cigarettes; M = Mean; No FS/FI = Missing Food Security Data

^b^Percentage across Participant Characteristic

^c^Percentage across Food Security Statuses by Participant Characteristic Group

Bivariate regression analyses (Table 3*)* that analyzed the association between food insecurity and heart disease risk factors without adjustment for covariates determined that food insecurity was positively associated with diabetes (B = 0.064, 95% CI = 0.011, 0.117; *p* = 0.02), cigarette smoking (B = 0.324, 95% CI = 0.226, 0.421; *p* < 0.0001), and BMI (B = 1.018, 95% CI = 0.231, 1.806; *p* = 0.02); negatively associated with sedentary time (B = -0.974, 95% CI = -1.574, -0.374; *p* = 0.004); and not significantly associated with high blood pressure (B = 0.065, 95% CI = -0.032, 0.163; *p* = 0.17) or high cholesterol (B = -0.030, 95% CI = -0.082, 0.022; *p* = 0.24).

Multivariable regression analyses (Table 3*)* that analyzed the association between food insecurity and heart disease risk factors adjusted for covariates demonstrated that food insecurity was positively associated with high blood pressure (B = 0.104, 95% CI = 0.001, 0.207; *p* = 0.04), diabetes (B = 0.087, 95% CI = 0.031, 0.143; *p* = 0.006), cigarette smoking (B = 0.239, 95% CI = 0.115, 0.363; *p* = 0.002); negatively associated with sedentary time (B = -0.765, 95% CI = -1.352, -0.179; *p* = 0.02); and not associated with high cholesterol (B = 0.010, 95% CI.= -0.040, 0.061; *p* = 0.65) or BMI (B = 0.888, 95% CI = -0.028, 1.804; *p* = 0.06).

**Table 3:** Regression estimates for the associations between food insecurity and heart disease risk factors among U.S. Adults ages 40 years and older (*n* = 3676)

Outcomes	Estimate	Standard Error	95% CI^a^	*p* Value
**High Blood Pressure**				
Unadjusted^b^	0.065	0.046	-0.032, 0.163	0.17
Adjusted^c^	0.104	0.046	0.001, 0.207	**0.04**
**High Cholesterol**				
Unadjusted^b^	-0.030	0.024	-0.082, 0.022	0.24
Adjusted^c^	0.010	0.023	-0.040, 0.061	0.65
**Diabetes**				
Unadjusted^b^	0.064	0.025	0.011, 0.117	**0.02**
Adjusted^c^	0.087	0.025	0.031, 0.143	**0.006**
**Cigarette Smoking**				
Unadjusted^b^	0.324	0.046	0.226, 0.421	**< 0.0001**
Adjusted^c^	0.239	0.056	0.115, 0.363	**0.002**
**Sedentary Time**				
Unadjusted^b^	-0.974	0.280	-1.574, -0.374	**0.004**
Adjusted^c^	-0.765	0.263	-1.352, -0.179	**0.02**
**Body Mass Index**				
Unadjusted^b^	1.018	0.367	0.231, 1.806	**0.02**
Adjusted^c^	0.888	0.411	-0.028, 1.804	0.06

^**a**^CI = Confidence Interval

^**b**^Unadjusted results were derived from bivariate linear regression or logistic regression analyses

^**c**^Adjusted results were derived from multivariable regression analyses using General Linear Models that statistically adjusted for Age, Gender, Race/Ethnicity, and Income

### Moderation analyses

The moderation of gender on the association between food insecurity and heart disease risk factors (Table 4) showed that women who were food insecure had significantly greater BMI (B = 2.284, 95% CI = 0.383, 4.186; *p* = 0.02) than men who were food insecure. In contrast, moderation analyses determined that the associations between food insecurity and high blood pressure (B = 0.071, 95% CI = -0.051, 0.192; *p* = 0.22), high cholesterol (B = 0.131, 95% CI = -0.004, 0.266; *p* = 0.06), diabetes (B = -0.007, 95% CI = -1.000, 0.086; *p* = 0.88), cigarette smoking (B = -0.124, 95% CI = -0.354, 0.105; *p* = 0.25), and sedentary time (B = 0.033, 95% CI = -1.635, 0.069; *p* = 0.94) did not significantly differ between men and women.

**Table 4:** Interaction effects of gender and food security status on cardiovascular disease risk factors among U.S. Adults ages 40 years and older (*n* = 3676)

Outcomes^a^	Estimate	Standard Error	95% CI^b^	*p* Value
**High Blood Pressure**				
Women	0.071	0.054	-0.051, 0.192	0.22
Men	–	–	–	–
**High Cholesterol**				
Women	0.131	0.060	-0.004, 0.266	0.06
Men	–	–	–	–
**Diabetes**				
Women	-0.007	0.041	-0.100, 0.086	0.88
Men	–	–	–	–
**Cigarette Smoking**				
Women	-0.124	0.101	-0.354, 0.105	0.25
Men	–	–	–	–
**Sedentary Time**				
Women	0.033	0.433	-1.635, 0.069	0.94
Men	–	–	–	–
**Body Mass Index**				
Women	2.284	0.841	0.383, 4.186	**0.02**
Men	–	–	–	–

^**a**^Outcomes were derived from multivariable regression analyses using General Linear Models that statistically adjusted for Age, Race/Ethnicity, and Income

^**b**^CI = Confidence Interval

The moderation of race/ethnicity on the association between food insecurity and heart disease risk factors (Table 5) showed that Asian adults with food insecurity (B = -0.164, 95% CI = -0.320, -0.008; *p* = 0.04) and Black adults with food insecurity (B = -0.153, 95% CI = -0.303, -0.002; *p* = 0.04) had lower rates of high blood pressure than White adults with food insecurity. Latino adults with food insecurity had lower rates of high cholesterol (B = 0.168, 95% CI = 0.001, 0.335; *p* = 0.04) than White adults with food insecurity. Black adults with food insecurity (B = -0.246, 95% CI = -0.463, -0.029; *p* = 0.03) and Latino adults with food insecurity (B = -0.380, 95% CI = -0.620, -0.140; *p* = 0.006) had lower rates of cigarette smoking than their White counterparts. No differences in diabetes, sedentary time, or BMI were detected across race/ethnicity categories among adults who were food insecure.

**Table 5:** Interaction effects of race/ethnicity and food security status on cardiovascular disease risk factors among U.S. Adults ages 40 years and older (*n* = 3676)

Outcomes^a^	Estimate	Standard Error	95% CI^b^	*p* Value
**High Blood Pressure**				
Asian	-0.164	0.069	-0.320, -0.008	**0.04**
Black	-0.153	0.067	-0.303, -0.002	**0.04**
Latino	-0.062	0.065	-0.209, 0.086	0.37
Other^c^	-0.104	0.099	-0.329, 0.121	0.32
White	–	–	–	–
**High Cholesterol**				
Asian	0.096	0.092	-0.112, 0.304	0.32
Black	0.019	0.060	-0.117, 0.155	0.76
Latino	0.168	0.074	0.001, 0.335	**0.04**
Other^c^	0.037	0.108	-0.208, 0.282	0.74
White	–	–	–	–
**Diabetes**				
Asian	-0.026	0.069	-0.182, 0.130	0.72
Black	-0.008	0.037	-0.092, 0.076	0.84
Latino	-0.018	0.037	-0.102, 0.067	0.65
Other^c^	0.008	0.110	-0.240, 0.257	0.94
White	–	–	–	–
**Cigarette Smoking**				
Asian	-0.368	0.175	-0.763, 0.027	0.06
Black	-0.246	0.096	-0.463, -0.029	**0.03**
Latino	-0.380	0.106	-0.620, -0.140	**0.006**
Other^c^	-0.103	0.090	-0.307, 0.102	0.29
White	–	–	–	–
**Sedentary Time**				
Asian	-0.534	0.554	-1.786, 0.719	0.36
Black	-0.109	0.392	-0.996, 0.777	0.79
Latino	-0.016	0.698	-1.594, 1.562	0.98
Other^c^	0.015	0.746	-1.673, 1.702	0.99
White	–	–	–	–
**Body Mass Index**				
Asian	0.456	0.841	-1.445, 2.357	0.60
Black	1.009	1.090	-1.458, 3.475	0.38
Latino	-0.018	1.133	-3.039, -0.180	0.99
Other^c^	0.650	1.897	-3.642, 4.943	0.74
White	–	–	–	–

^**a**^Outcomes were derived from multivariable regression analyses using General Linear Models that statistically adjusted for Age, Gender, and Income

^**b**^CI = Confidence Interval

^c^Other race/ethnicity category includes American Indian or Alaska Native, Native Hawaiian or Pacific Islander, and Mixed Race

## Discussion

This investigation offered valuable insights regarding the association between food insecurity and CVD risk factors among U.S. adults. Food insecurity was positively associated with high blood pressure, diabetes, cigarette smoking, and BMI, while it was negatively associated with sedentary time. These outcomes generally substantiate and expand upon those from past studies that found food insecurity to be a harmful contributor to CVD risk.

Examination into the unique moderating effects of gender and race/ethnicity provided noteworthy evidence highlighting disparities in CVD risk factors among U.S. adults with food insecurity. Women who were food insecure had higher BMI scores than men who were food insecure. White adults who were food insecure were more likely to have high blood pressure and cigarette smoking than adults of other races who were also food insecure. Meanwhile, Latino adults who were food insecure had greater rates of high cholesterol than White adults who were food insecure.

Regarding the CVD risk factor of high cholesterol, outcomes from this study align with results from past research among a population-based sample of midwestern U.S. adults aged 21–74 years old that found no association between food insecurity and high cholesterol [35], as the present study also found association between food insecurity and high cholesterol among this national sample of U.S. adults aged 40 years or older. Outcomes from this study differ from results produced by prior research studies that reported no significant association between food insecurity and sedentary time among U.S. adults aged 18–65 years old [31], as the present study found that U.S. adults with food insecurity who were 40 years of age or older had lower sedentary time than their food secure counterparts. Age differences may be a factor for these differing findings, as the prior research included younger adults while this study did not. Further investigations are needed to ascertain whether age moderates the negative association between food insecurity and sedentary time among U.S. adults, as an insufficient number of studies have examined these associations.

Identifying gender differences in the associations between food insecurity and CVD risk factors was a primary objective in the present study. Findings from the present study filled a gender-specific knowledge gap by detecting no significant differences between men and women in the association between food insecurity and high blood pressure. An additional novel finding in this study was the result of no significant gender differences in the association between food insecurity and sedentary time. While the gender-specific outcomes for these two CVD risk factors were both non-significant, such findings merit continued investigation.

The lone significant finding from the evaluation of gender differences in the association between food insecurity and CVD risk factors was for the outcome of BMI. The “food insecurity-obesity paradox” was conceptualized over a decade ago in response to the seemingly paradoxical positive association between food insecurity and BMI that refuted a prevailing assumption about how people who were food insecure should not be overweight or obese due to their insufficient access to food [42]. Similar to what was originally reported in the seminal food insecurity-obesity article [42], findings from the present study suggested a significant positive association between food insecurity and BMI among women but not among men. Since the preliminary portrayal of this purported paradox, much research has been conducted to both confirm and clarify the once peculiar positive association between food insecurity and obesity [43]. Outcomes from one recent cross-sectional study among a sample of over 25,000 U.S. adults helped to corroborate a gender-specific link between food insecurity and BMI, as women with food insecurity had significantly greater BMI than their food secure counterparts while BMI did not significantly differ by food security status among men [44]. Another cross-sectional study that examined whether the food insecurity-obesity paradox among women varied by race/ethnicity determined that categories a greater percentage of women with food insecurity were overweight or obese compared to women who were food secure across all racial/ethnic, while no link between food insecurity and overweight or obesity was again reported for men [29]. Much uncertainty remains concerning the underlying mechanisms within the association between food insecurity and BMI among women, as research has sought to determine why food insecurity is related to obesity among women but not men without arriving at a conclusive understanding [45]. 

Gender-specific outcomes in this study regarding the associations between food insecurity and high cholesterol, diabetes, and cigarette smoking all differed from findings produced by prior research. Outcomes from the present study suggested there were no gender differences in the association between food insecurity and high cholesterol. Such findings differ from those produced in prior cross-sectional research that highlighted how food insecurity was linked to high cholesterol in women but not men [33]. This study also did not detect gender differences in the association between food insecurity and diabetes. These findings differ from those yielded by a past cross-sectional study that identified a positive association between food insecurity and diabetes among women but not men [46] along with outcomes from other cross-sectional research that determined a positive association among Latino women but not Latino men [47]. Lastly, this study found no gender differences in the association between food insecurity and cigarette smoking. These results differ from those produced in a previous cross-sectional study among U.S. adults that demonstrated how food insecurity was associated with increased smoking among women but not among men [48]. Behavioral and social mechanisms that can modify the association between food insecurity and CVD risk factors among men and women should be considered as potential explanations for these contradictory findings. These associations must be further examined with longitudinal research methods that permit the tracking of changes in food insecurity and CVD outcomes over time.

Separate moderation outcomes from the present study also expanded, supported, or challenged existing evidence for the extent to which race/ethnicity moderated the associations between food insecurity and the six CVD risk factors of interest. This study expanded the field of food insecurity research by generating novel insights highlighting racial/ethnic health disparities that are critical for better understanding the association between food insecurity and CVD. Prior cross-sectional research had measured rates of hypertension among U.S. adults with food insecurity by race/ethnicity but only reported the prevalence of hypertension and did not determine whether the prevalence significantly differed across races/ethnicities [49]. The present study expands this knowledge with evidence demonstrating racial/ethnic differences in the association between food insecurity and high blood pressure. Furthermore, a previous cross-sectional study highlighted the prevalence of obesity, hypertension, and diabetes across the four food security categories for each racial/ethnic group without determining whether the association between food insecurity and those three CVD risk factors differed by race/ethnicity [50]. While the present study did not explicitly measure obesity and hypertension, greater understandings were achieved regarding racial/ethnic differences in the associations between food insecurity and high blood pressure, diabetes, and BMI.

The present study produced outcomes for racial/ethnic differences in the association between food insecurity and CVD risk factors that challenge evidence from several past studies. While the present study found White adults with food insecurity had increased rates of high blood pressure compared to Asian and Black adults with food insecurity, a cross-sectional study that examined racial/ethnic differences in the prevalence of hypertension among U.S. adults with food insecurity found that Hispanic adults with food insecurity had a greater prevalence of hypertension than their Black and White counterparts [49]. The reason for these differing results could be due to geographic differences in the participant sample since the present study has a national sample of adults and the previous study only sampled from 12 U.S. states. This supposition raises a consideration for geographic (e.g., region, state) differences in the association between food insecurity, high blood pressure, and other CVD risk factors. Separately, results in the present study related to diabetes contradict outcomes from a cross-sectional study among U.S. adults aged 55 years or older that found that Asian, Black and Mexican American adults with food insecurity had greater odds of diabetes than White adults with food insecurity [46]. Sampling differences could again explain the contradicting evidence since the present study sampled adults who were 40 years of age or older and the prior study sampled adults who were 55 years of age or older.

Novel findings from the present study filled an important knowledge gap concerning racial/ethnic differences in the association between food insecurity and high cholesterol among U.S. adults. No known studies had previously evaluated racial/ethnic differences in the association between food insecurity and high cholesterol, and outcomes from this study revealed that Latino adults who were food insecure had greater rates of high cholesterol than White adults with food insecurity. The present study filled a separate race/ethnicity-specific knowledge gap by identifying how both Black adults with food insecurity and Latino adults with food insecurity had lower rates of cigarette smoking than their White counterparts. Additionally, no known studies had explored racial/ethnic differences in the association between food insecurity and sedentary time, and the present study found no significant racial/ethnic differences in sedentary time across U.S. adults with food insecurity.

These novel findings strengthen and expand the knowledge base for the association between food insecurity and CVD risk factors. To promote health equity by addressing racial/ethnic disparities in CVD, additional research is needed to establish a consensus for the extent to which race/ethnicity moderates the associations between food insecurity and these CVD risk factors. Furthermore, greater consideration of behavioral and social differences between racial/ethnic groups should be accounted for as modifiers of these associations. High-risk groups such as women and racial/ethnic minorities who are food insecure can benefit from specially tailored Food is Medicine (i.e., produce prescriptions, medically tailored meals, etc.) interventions that are informed by these findings and geared toward preventing CVD with culturally acceptable approaches.

### Strengths and limitations

This study contained several strengths. First, the study participants consisted of a large, national sample of U.S. adults aged 40 years and older. The large sample size paired with the sampling weights used in the analyses permitted the performance of precise cross-sectional analyses among a nationally representative sample that produced reliable and valid results. Second, novel information was produced detailing non-significant gender differences in the association between food insecurity and the CVD risk factors of high blood pressure and sedentary time. Additional novel information was acquired for racial/ethnic differences in the association between food insecurity and both high cholesterol and sedentary time. Third, the present study expanded knowledge from prior studies on racial/ethnic differences within the association between food insecurity and CVD risk factors by testing for and reporting statistical significance values for the outcomes of high blood pressure, diabetes, and BMI. Fourth, the research objectives in this study were achieved which resulted in critical insights detailing the detrimental association between food insecurity and CVD, the leading cause of death among U.S. adults. These insights can inform CVD prevention efforts that are urgently needed for U.S. adults with food insecurity.

The present study also had various limitations. First, the cross-sectional research design prevented the study results from being interpreted with a causal or temporal perspective. Food insecurity and the CVD risk factors were respectively posited as the independent variable and dependent variables in this study, but the single timepoint of the NHANES data that was accessed cannot be analyzed to demonstrate a causal effect of food insecurity on the CVD risk factors. Second, the dataset analyzed in this study was accessed from the CDC’s archive of NHANES data, which meant that researchers in this study had no control over how the outcomes of interest were measured. The archival nature of the dataset introduced bias by not allowing investigators within this study to account for other potential confounders that were not measured by the NHANES survey. A strategy used to reduce this bias included the statistical adjustment of covariates by controlling for sociodemographic variables that could have otherwise confounded the associations between food insecurity and CVD risk factors. Nonetheless, residual confounding from unmeasured confounders may have limited the precision of the statistical analyses results. Third, several outcome measures for the CVD risk factors combined hereditary and non-hereditary outcomes (e.g., diabetes) or included time ranges for diagnoses that spanned the entire life course instead of a more recent history (e.g., blood pressure, cholesterol). Including a separate variable to type 1 diabetes and type 2 diabetes would have allowed for a more precise evaluation of the association between food insecurity and diabetes. The outcome measures for high blood pressure, high cholesterol, and diabetes could have been exclusive to adulthood, or a more recent history, to yield outcome estimates that were more temporally proximal to each other. Fourth, the data used for this study was entirely self-report, which introduced threats to measurement validity by requiring participants to accurately remember (recall bias) and truthfully report (social desirability bias) their food insecurity and CVD risk factor outcomes. Self-report is a less preferred method of measuring outcomes when they can be feasibly measured with objective approaches like a height/weight scale (BMI) and a wearable fitness tracker (sedentary time). Although, self-report essential for measuring psychosocial variables, like food insecurity, that cannot otherwise be measured.

### Future directions

The several significant associations that were detected in this study strengthened and expanded the evidence linking food insecurity to various behavioral and health outcomes that act as CVD risk factors. While the findings from this cross-sectional study cannot identify food insecurity as a predictor of CVD risk, it can be posited that food insecurity is significantly associated with multiple biomarkers, medical conditions, and behaviors that are risk factors for CVD. Therefore, longitudinal studies are needed to track U.S. adults with varying food security statuses to expand findings from this study toward determining whether food insecurity has a causal effect on CVD risk factors over time. Separately, differences in the associations between food insecurity and CVD risk factors across age groups from middle-to-older adulthood need to be better understood. Furthermore, mediation studies should test the extent to which individual CVD risk factors mediate the longitudinal effect of food insecurity on CVD.

## Conclusions

Food insecurity is associated with numerous CVD risk factors, and such associations are influenced by the person-level characteristics of gender and race/ethnicity. Outcomes from the present study could be used to inform future longitudinal studies that are needed to examine whether food insecurity worsens CVD risk factors and CVD outcomes themselves. Such longitudinal evidence could then be used to inform the design of prevention and intervention efforts for both food insecurity as a harmful social condition and CVD as the primary cause of death among U.S. adults.

## Data Availability

The datasets used and analyzed during the current study are available from the corresponding author on reasonable request.

## Abbreviations

AFSSM: Adult Food Security Survey Module
B: Beta Coefficient
BMI: Body Mass Index
BP: Blood Pressure
CAD: Coronary Artery Disease
CDC: Centers for Disease Control and Prevention
CI: Confidence Interval
CVD: Cardiovascular Disease
GLM: General Linear Model
KG: Kilograms
M: Mean
MTR: Meters
NHANES: National Health and Nutrition Examination Survey
US: United States
USDA: United States Department of Agriculture
SD: Standard Deviation
SE: Standard Error
